# Glatiramer Acetate and Nanny Proteins Restrict Access of the Multiple Sclerosis Autoantigen Myelin Basic Protein to the 26S Proteasome

**DOI:** 10.1155/2014/926394

**Published:** 2014-09-08

**Authors:** Ekaterina Kuzina, Anna Kudriaeva, Ivan Smirnov, Michael V. Dubina, Alexander Gabibov, Alexey Belogurov

**Affiliations:** ^1^Shemyakin-Ovchinnikov Institute of Bioorganic Chemistry, Russian Academy of Sciences, V-437, Moscow 117871, Russia; ^2^Chemistry Department, Lomonosov Moscow State University, Moscow 119991, Russia; ^3^Kazan Federal University, Kazan, Republic of Tatarstan 420008, Russia; ^4^Nanotechnology Research and Education Centre RAS, St. Petersburg Academic University, St. Petersburg 194021, Russia; ^5^Institute of Gene Biology, Russian Academy of Sciences, Moscow 117334, Russia

## Abstract

We recently showed that myelin basic protein (MBP) is hydrolyzed by 26S proteasome without ubiquitination. The previously suggested concept of charge-mediated interaction between MBP and the proteasome led us to attempt to compensate or mimic its positive charge to inhibit proteasomal degradation. We demonstrated that negatively charged actin and calmodulin (CaM), as well as basic histone H1.3, inhibit MBP hydrolysis by competing with the proteasome and MBP, respectively, for binding their counterpart. Interestingly, glatiramer acetate (GA), which is used to treat multiple sclerosis (MS) and is structurally similar to MBP, inhibits intracellular and *in vitro* proteasome-mediated MBP degradation. Therefore, the data reported in this study may be important for myelin biogenesis in both the normal state and pathophysiological conditions.

## 1. Introduction

Myelin basic protein (MBP) is one of the major autoantigens in the pathogenesis of multiple sclerosis (MS) [1] and experimental autoimmune encephalomyelitis [2]—animal model of MS. MBP and its peptides have been extensively studied as important components of the autoimmune pathology of the central nervous system (CNS). A number of MBP peptides were found to be strongly associated with MHC class II [3, 4] in MS patients. Although MS is thought to be mainly a CD4+ T cell-mediated disease, myelin-specific cytotoxic lymphocytes, which recognize MHC I-restricted MBP peptides, can lyse human oligodendrocytes in cell culture [5] and cause severe EAE in mice [6]. The fragments of intracellular proteins that are presented on the MHC class I molecules are generated mainly by the multicatalytic proteinase complex—a 26S proteasome [7]. The majority of cellular proteins are degraded by the 26S proteasome in a ubiquitin-dependent manner [8]. The polyubiquitin chains interact with the 19S regulatory particle, which catalyzes the deubiquitination and denaturation of the substrate and its translocation into the 20S catalytic chamber [9, 10]. Interestingly, recent data indicate that proteasome substrates may be polymonoubiquitinated [11] or even modified by single ubiquitin moieties [12]. Moreover, the number of proteins, such as ornithine decarboxylase [13] and p21 [14], can be degraded by the 26S proteasome without ubiquitination in an ATP-dependent manner [15]. Uncapped 20S proteasome particles are also active in the degradation of either completely or regionally disordered nonubiquitinated proteins, such as*α*-synuclein [16] and p53 [17]. Recently we have shown that the 26S proteasome can hydrolyze MBP at physiologically relevant concentrations without ubiquitination* in vitro* and in mature oligodendrocytes [18]. Therefore, proteasome-mediated MBP degradation, which generates myelin antigenic peptides [19, 20], is of a critical importance for the pathogenesis of CNS-related autoimmune diseases.

MBP is known to be highly flexible and intrinsically disordered [21], suggesting that electrostatics forces may primarily determine its interactions with other proteins. We have previously found that the ubiquitin-independent proteolysis of MBP seems to be charge-mediated, as 26S proteasome less efficiently degrades deiminated MBP bearing a decreased positive charge [18]. Therefore, the intracellular counterparts of MBP may restrict its accessibility to the 26S proteasome. Alternatively, proteins that mimic MBP may compete with proteasome for MBP binding. Intracellular MBP may bind Ca^2+^-activated calmodulin (CaM), actin, tubulin, and proteins containing SH3 domains. MBP is believed to be associated with the cytoskeleton and interacts with actin in oligodendrocytes* in vivo* [22]. The ability of MBP to polymerize actin depends on the net positive charge of the MBP molecule [23]. Full-length MBP is known to bind CaM, a highly acidic calcium sensor, under near-physiological conditions [24]. MBP is a major calcium-dependent CaM-binding protein in human brain white matter MBP, and CaM is colocalized in cultured myelin [25]. An 18.5-kDa MBP has been shown to bind to several SH3 domains, including that of Fyn, a member of the Src family of tyrosine kinases that is involved in a number of signaling pathways during CNS development [26]. The surface charge density of the Fyn-SH3 domain is negative, and the rate of its binding to MBP depends on the MBP net positive charge [27]. In the present study, we investigated whether the interaction of MBP or 26S proteasome with a number of charged proteins could interfere with ubiquitin-independent MBP degradation.

## 2. Materials and Methods

### 2.1. Proteins

MBP was prepared from bovine brains according to [28]. The obtained protein was purified by reverse phase HPLC on a C_4_ 10/250 column (Macherey-Nagel). Actin from porcine muscle, lysozyme from chicken egg, calmodulin from bovine brain, and BSA were obtained from Sigma. Recombinant histone H1.3 was obtained from* E. coli*, and recombinant human ubiquitin and recombinant human K48-tetraubiquitin were obtained from Boston Biochem. GA (Copaxone) is a commercially available drug from Teva; for the experiments, it was desalted into 20 mM Tris-HCl pH 7.5 using a HiTrap Desalt column (GE Healthcare Life Sciences).

### 2.2. Cultured Cells and Transfection Procedures

HEK293 cells were grown at 37°C and 5% CO_2_ in DMEM supplemented with 10% fetal calf serum and antibiotics (penicillin-streptomycin). The cells were transfected with the pBudCE4.1/EF-FLAG plasmid carrying human MBP or the human histone H1.3 sequence. The cDNA transfections were accomplished using Lipofectamine LTX with Plus reagent (Life Technologies). All of the procedures were performed according to the manufacturer's instructions.

### 2.3. Cycloheximide Chase Experiments

To study the proteasomal degradation of MBP and histone H1 in HEK293 cells, cycloheximide (100 *μ*g/mL) was added to transfected cells for the indicated times, and the cells were lysed using RIPA buffer (150 mM NaCl, 0.5% sodium deoxycholate, 50 mM Tris-HCl pH 8, 0.1% SDS, 1% NP-40, and protease inhibitors mixture). Protein lysates prepared from an equal number of cells were resolved via SDS-PAGE and blotted onto nitrocellulose membranes. MBP and histone H1 were visualized using an anti-FLAG antibody (A8592, Sigma-Aldrich). *β*-Actin was used as a loading control and detected using a specific antibody (sc-81178, Santa Cruz Biotechnology).

### 2.4. Purification of the Proteasome from Mouse Liver

Briefly, a BALB/c brain was homogenized using a Dounce homogenizer into three parts w/w lysis buffer containing 30 mM Tris-HCl (pH 7.5), 2 mM ATP, 1 mM EDTA, 5 mM MgCl_2_, 1 mM DTT, 10% glycerol, 150 mM NaCl, and a protease inhibitor cocktail. The prepared brain homogenate was subjected to three repeated freeze-thaw cycles, and further cell debris was removed via two consecutive centrifugations at 4°C (1,500 g for 20 min and 13,000 g for 30 min). Ammonium sulfate was added to the supernatant to 40% saturation, and the mixture was agitated for 40 min at 4°C. The precipitate was collected by centrifugation (13,000 g 10 min at 4°C), dissolved in buffer containing 20 mM Tris (pH 7.5), 10% glycerol, 150 mM NaCl, 1 mM ATP, 1 mM DTT, 1 mM EDTA, and 5 mM MgCl_2_, and loaded on a Superose 6 column (GE Healthcare Life Sciences). The fractions (1 mL each) were collected, and the proteasome activity was quantified using Suc-LLVY-MCA as a substrate. To distinguish between the activity related to the 20S proteasome and that related to the 26S proteasome, the assay was performed with or without 0.02% SDS. The buffer used to measure the activity of the proteasomes contained 20 mM Tris (pH 7.5), 1 mM ATP, 1 mM DTT, and 5 mM MgCl_2_. The fractions containing the 26S proteasome were subjected to ion-exchange chromatography on a MonoQ column using a NaCl gradient (275–1000 mM in 20 column volumes) in buffer containing 20 mM Tris (pH 7.5), 10% glycerol, 0.1 mM ATP, 1 mM DTT, and 0.1 mM EDTA. The fractions containing the 26S proteasome were dialyzed into storage buffer (25 mM Tris-HCl [pH 7.5], 1 mM DTT, 1 mM ATP, 5 mM MgCl_2_, and 10% glycerol). The concentration of proteasome was determined with a Bradford assay. For a long-term storage, up to 40% glycerol was added to the proteasome, and the purified proteasome was stored at −20°C for two months.

### 2.5. Native PAGE

Proteasome samples (200 ng) were loaded on a 4% gel (acrylamide: N,N′-methylenebisacrylamide 37.5 : 1, 180 mM Tris-borate buffer [pH 8.3], 5 mM MgCl_2_, 1 mM DTT, and 1 mM ATP). Electrophoresis was conducted for 1.5 h at 4°C and 180 V. The gels were soaked in buffer containing 20 mM Tris-HCl (pH 7.5), 5 mM MgCl_2_, 1 mM DTT, and 1 mM ATP supplemented with 100 mM Suc-LLVY-MCA for 10 min at 37°C and visualized on a Versa Doc Imaging system (Bio-Rad) using the trans-UV excitation and the 530BP emission filter.

### 2.6. Proteasome Ultracentrifugation

The 20S and 26S proteasomes from BALB/c mouse brains were separated by ultracentrifugation. The tissue was homogenized (Dounce homogenizer, Thomas Scientific) in 3 V buffer containing 20 mM Tris-HCl (pH 7.5), 10% glycerol, 150 mM NaCl, 1 mM EDTA, 5 mM MgCl_2_, 1 mM DTT, and protease inhibitor cocktail (Roche), and the homogenates were centrifuged (16,000 g, 4°C 30 min) to remove cell debris. To study MBP binding to the proteasome, the homogenates were incubated with purified bovine MBP in the presence of 1 *μ*M PS-341 for 30 min at 4°C. Further homogenates were separated by ultracentrifugation in 10% to 55% glycerol gradient in buffer containing 20 mM Tris-HCl (pH 7.5), 5 mM MgCl_2_, 1 mM DTT, and 1 mM ATP at 125,000 g for 18 h at 4°C. The proteasome activity in the resulting fractions was measured using Suc-LLVY-MCA as a substrate in the presence and absence of 1 *μ*M PS-341 and 0.02% SDS.

### 2.7. Association of MBP with 26S

Bovine MBP (1 *μ*g) and PS-341-pretreated 26S (3 *μ*g) were incubated for 1 h in 100 *μ*L of buffer containing 20 mM Tris-HCl (pH 7.5), 20% glycerol, 1 mM DTT, 1 mM ATP, 200 *μ*g/mL BSA, 0.1% NP-40, and 100 mM NaCl at 4°C. MBP-26S complexes were precipitated with the addition of rat monoclonal anti-MBP (ab7349, Abcam) or mouse polyclonal anti-hRpn10 antibodies (H00005710-B01P, Abnova), followed by incubation with the protein G-sepharose. The resulting immunoprecipitates were subjected to Western blotting analysis and further stained for MBP (ab77895, Abcam) and hRpn2 (ab21638, Abcam).

### 2.8. *In Vitro* Protein Degradation by Proteasome

The proteasome samples were mixed with bovine MBP and one of tested proteins (actin, CaM, histone H1.3, GA, lysozyme, BSA, GST, Ub, and K48-Ub_4_) in buffer containing 20 mM Tris-HCl (pH 7.5), 5 mM MgCl_2_, 1 mM DTT, and 1 mM ATP and incubated for 2 h at 37°C. The MBP concentration in the reaction mixture was 90 ng/*μ*L, the proteasome concentration was 50 ng/*μ*L (proteasome to substrate 1 : 250), and the concentrations of tested proteins were 60, 180, 360, and 600 ng/*μ*L.

### 2.9. Chymotrypsin-Like Proteasome Activity Assay

26S proteasome (0.1 *μ*g/*μ*L) was mixed with Suc-LLVY-MCA substrate (25 *μ*M) in buffer containing 20 mM Tris-HCl (pH 7.5), 5 mM MgCl_2_, 1 mM DTT, and 1 mM ATP with or without the tested protein (1 *μ*g/*μ*L). The rate of hydrolysis was measured on a Varioskan Flash plate fluorimeter (Thermo Scientific) at an excitation of 360 nM and emission of 460 nM.

### 2.10. Surface Plasmon Resonance

The SPR measurements were performed on a Biacore T200 apparatus. The ligands were immobilized on CM4 chips (~1,500 RU) using an amino-coupling kit according to the manufacturer's instructions. All of the analyte binding measurements were performed with HBS-EP+ as the continuous running buffer at 25°C. Actin, GA, and histone H1.3 were injected at a concentration of 5.0 *μ*M and a flow rate of 25 *μ*L/min for 200 s. The binding sensorgrams were analyzed using the BIAevaluation Software.

## 3. Results

**Figure 1:**
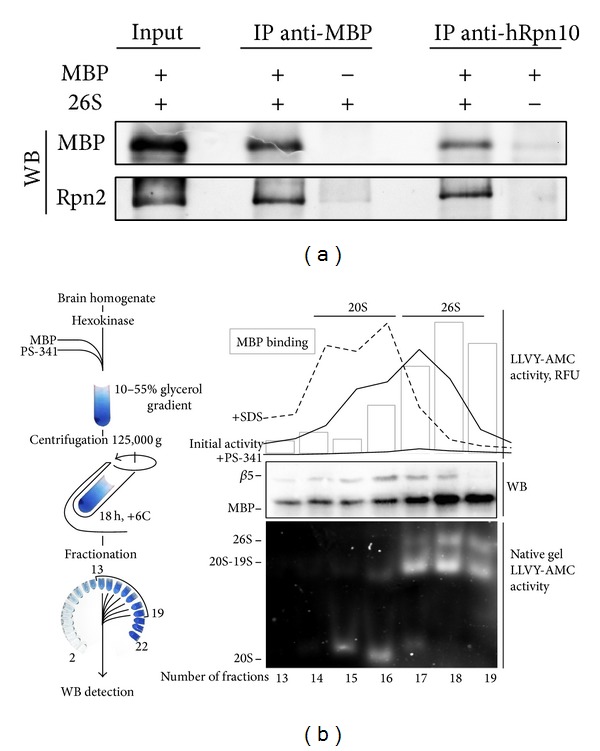
(a) Western blotting analysis of the immunoprecipitation of the 26S proteasome complex with bovine MBP using monoclonal anti-MBP or anti-hRpn10 antibodies. (b) PS-341-treated 20S and 26S proteasomes from BALB/c brain homogenate were preincubated with MBP, separated by ultracentrifugation, and analyzed for bound MBP by Western blotting, as indicated. The presence or absence of the 19S regulatory particle verified by Western blotting for *β*5 proteasome subunit, native PAGE, and LLVY-AMC activity profiles in the presence or absence of SDS and PS-341.

To test for a possible direct interaction of MBP with 26S proteasome, we incubated PS-341-treated 26S proteasome with MBP and further immunoprecipitated proteins using either anti-MBP or anti-hRpn10 antibodies. In both cases, the eluates contained MBP and 26S proteasome, whereas no cross-reactivity of anti-hRpn10 and anti-MBP antibodies with MBP and the proteasome, respectively, was observed, suggesting that the proteasome binds MBP* in vitro* (Figure 1(a)). The 26S proteasome consists of two subparticles, namely, a hollow barrel-shaped 20S particle that contains multiple proteolytic sites and a regulatory 19S subunit that is required to recognize the polyubiquitination signal [9]. The fractionation of proteasomes mixed with MBP by glycerol gradient centrifugation demonstrated that MBP was coeluted with 26S but not with 20S proteasomes (Figure 1(b)). This finding agrees with our previous observations suggesting that MBP-proteasome interaction is charge-mediated, as the acidic isoelectric point of the majority of 19S regulator subunits is below 6 (pI values of 19S subunits of eukaryotic proteasome are listed in [29]).

**Figure 2:**
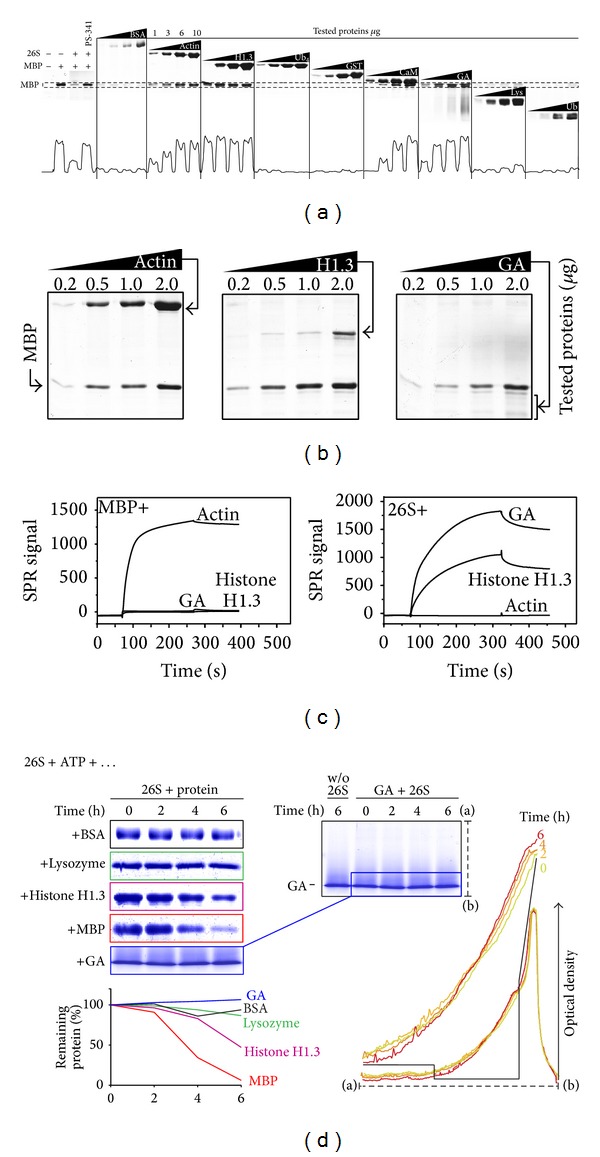
(a and b) Degradation of bovine MBP by 26S proteasome in the presence of tested proteins (1–10 *μ*g) as indicated. The bottom curve on panel (a) represents the densitometry analysis of the remaining MBP (dashed area on top). (c) Sensorgrams from SPR measurements for the interaction between GA, histone H1.3, CaM, and immobilized MBP (left panel); 26S proteasome and immobilized actin, GA, and histone H1.3 (right panel). (d) Degradation of BSA, lysozyme, histone H1, GA, and MBP by 26S proteasome in presence of ATP as monitored by PAGE. The percentage of protein remaining was calculated as the ratio of protein at the indicated time-points relative to the initial protein. The insertion shows the overlaid densitometry profiles of GA samples incubated with the 26S proteasome.

To obtain further details on the mechanism of proteasomal MBP degradation, we further made an attempt to intercept MBP before it could reach the proteasome or, alternatively, mimic MBP to compete with it for proteasome binding. To this end, we selected a number of proteins (Table 1) that could potentially interfere with the hydrolysis of MBP by proteasome: (i) actin [30] and CaM [31], which are known to bind MBP* in vitro* and* in vivo*; (ii) the anti-MS drug glatiramer acetate (GA), which is structurally similar to MBP [32]; (iii) positively charged and intrinsically disordered histone H1.3 [33]; (iv) mono- and tetra-ubiquitin (Ub, Ub_4_), which can bind the ubiquitin receptors of the 19S regulator [34, 35]; (v) the slightly acidic globular proteins GST and BSA and basic lysozyme with compact globular structure which were used as controls. The hydrolysis of MBP was monitored in a ubiquitin-free* in vitro* system containing purified 26S proteasome, ATP, test proteins, and none of the components of the ubiquitination system. The rate of MBP hydrolysis was analyzed by SDS-PAGE (Figure 2(a)). Neither Ub_4_ nor mono-Ub, which, respectively, binds to the ubiquitin interaction motifs of Rpn10 [34] and N-terminal segment of Rpn13 [35], significantly changes the rate of MBP hydrolysis by the proteasome. This result suggests that the Ub-binding domains are not involved in MBP recognition by the 19S regulator. GST, BSA, and lysozyme did not change the rate of MBP hydrolysis, while actin, histone H1.3, CaM, and GA obviously inhibited MBP degradation (Figures 2(a) and 2(b)). We further tested the ability of actin, GA, and histone H1.3 to bind to MBP and the 26S proteasome. According to the SPR measurements, MBP interacted with negatively charged actin but not with GA and histone H1.3 (Figure 2(c), left panel). In contrast, the 26S proteasome bound GA and histone H1.3, but not actin (Figure 2(c), right panel). Therefore, proteins with detected inhibitory activity were evidently divided into two subgroups, particularly those that bind MBP and those that bind the 26S proteasome. Interestingly, among proteasome binders, GA itself was resistant to proteasomal hydrolysis, whereas histone H1.3 was degraded by 26S proteasome to some extent (Figure 2(d), left panel). According to the precise densitometry analysis, the observed migration of the GA molecular weight distribution to the less heavy masses is explained by the dynamic processes of aggregation/disaggregation rather than by 26S-mediated hydrolysis (Figure 2(d), right panel).

**Figure 3:**
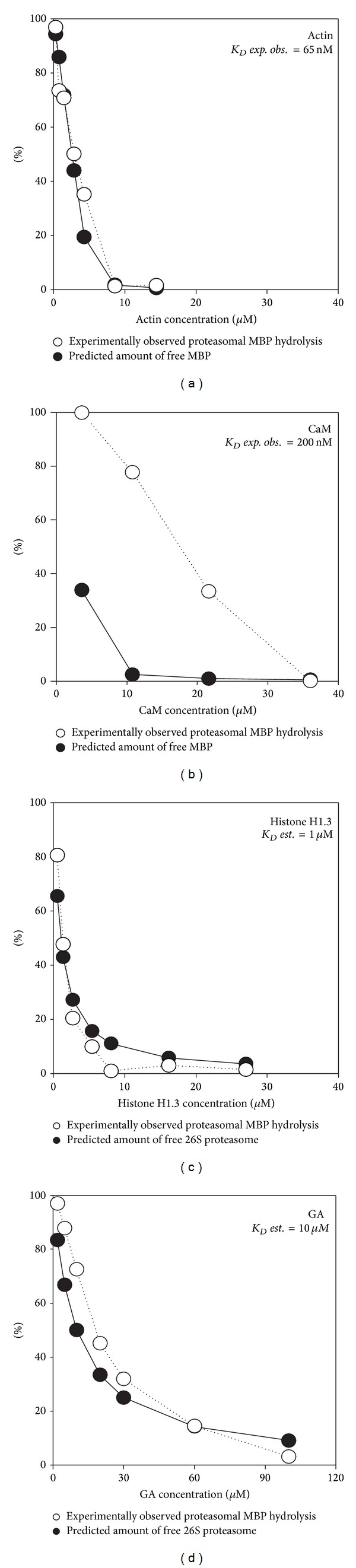
Plots represent the percentage of MBP hydrolysis (opened circles) by 26S proteasome in the presence of the indicated concentrations of actin (a), CaM (b), histone H1.3 (c), and GA (d). The theoretical percentage of uncomplexed MBP (filled circles on panels (a) and (b)) was calculated using previously reported *K*_*D*_ (*exp.obs.*) values for the MBP-actin and MBP-CaM interactions, respectively. The theoretical percentage of histone H1.3- or GA-bound 26S proteasome (filled circles on panels (c) and (d)) was calculated using an asymptotic approximation of the percentage of MBP hydrolysis, assuming that the bound proteasome cannot degrade MBP (theoretical *K*_*D*_ *est.*).

The extent of MBP hydrolysis in the presence of actin, CaM, histone H1.3, and GA (Figures 2(a) and 2(b)), as estimated by densitometry analysis, was plotted as a function of the concentration of inhibitory proteins (Figure 3). Furthermore, the experimentally observed extent of MBP hydrolysis in the presence of different concentrations of actin and CaM was compared with the theoretical amount of uncomplexed MBP calculated using previously reported dissociation constants (*K*_*D*_) for the MBP-actin (Figure 3(a)) and MBP-CaM (Figure 3(b)) interactions. The reversed task was accomplished using experimental curves that represent the inhibition of proteasome-mediated MBP proteolysis by GA and histone H1.3. Using the asymptotic approximation of the percentage of MBP hydrolysis, we estimated the theoretical *K*_*D*_ of 26S-histone H1.3 (Figure 3(c)) and 26S-GA (Figure 3(d)) interaction by assuming that bound 26S proteasome cannot degrade MBP.

**Figure 4:**
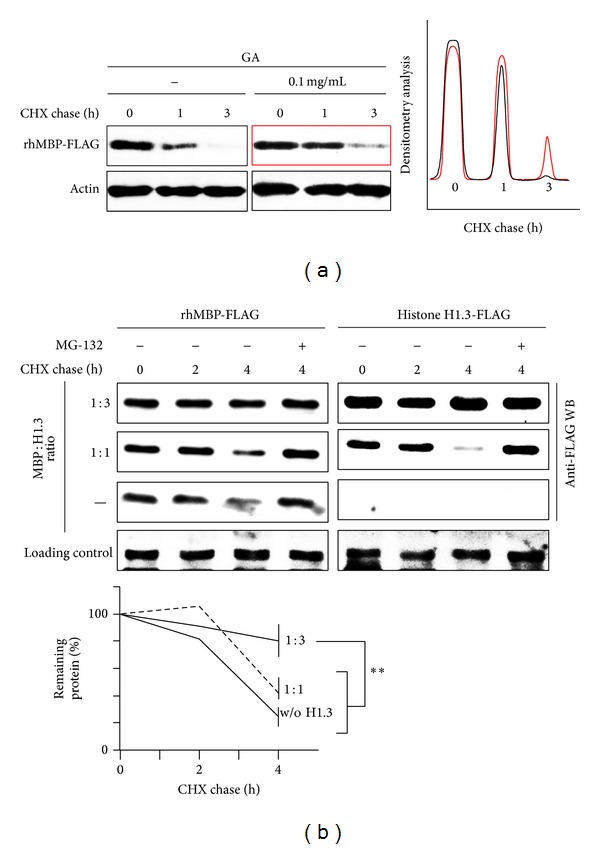
(a) HEK293 cells were transfected with cDNA coding for human MBP with C-terminal FLAG epitope (hMBP-FLAG). After 4 h, the cells were incubated for an additional 20 h with or without GA, as indicated. The cells were then subjected to a cycloheximide chase followed by Western blotting. (b) The HEK293 cells were transfected with cDNAs coding for rhMBP-FLAG along with cDNA coding for the human histone H1.3-FLAG, as indicated. After 24 h, the cells were subjected to a cycloheximide chase followed by western blotting analysis. The percentage of protein remaining was calculated as the ratio of protein at the indicated time-points relative to the initial protein. The data are represented as the mean ± SEM from three separate experiments. ∗∗ denotes *P* < 0.01.

We monitored degradation of MBP in HEK293 cells to determine if the discovered inhibitory effects may be observed* in vivo*. To this end, we transiently transfected HEK293 cells with cDNA coding for human MBP (hMBP) and either cotransfected cDNA coding for human histone H1.3 or added GA into the culture medium. The cells were treated with cycloheximide (CHX), harvested at the indicated time-points, and further subjected to western blotting analysis. Both GA (Figure 4(a)) and histone H1.3 (Figure 4(b)) decreased the intracellular proteasome-mediated MBP hydrolysis. Similar to the* in vitro* assays, transiently transfected histone H1.3 was partially degraded by the proteasome in HEK293 cells.

## 4. Discussion

Here, we showed that CaM and actin protect MBP from proteasomal hydrolysis. Previous findings indicate that the dissociation constant (*K*_*D*_) of MBP-CaM interaction is approximately 200 nM [36] for recombinant murine MBP or 148 nM for MBP from porcine brain [25], as determined by SPR. The interaction of MBP with actin is characterized by a *K*_*D*_ of 66.6 nM for G-actin or 65.3 nM for F-actin [30]. Importantly, the rate of* in vitro* MBP hydrolysis in the presence of actin agrees with the predictions of concentration of “free” MBP based on previously reported dissociation constants (Figure 3(a)). We failed to correlate the amount of unbound MBP with the rate of CaM-mediated inhibition of proteasomal MBP degradation. We further reasoned that this observation is due to different mechanisms of MBP binding. MBP-actin interaction is believed to be charge-mediated, as the ability of MBP to polymerize actin depends on the net positive charge of the MBP molecule—the rate and extent of actin polymerization induced by 18.5 kDa MBP charge isomers correlate with the charge reduction caused by posttranslational modifications [23]. The MBP in MBP-actin assemblies is structurally heterogeneous but gains ordered secondary structure elements (both*α*-helices and *β*-sheets), particularly in the terminal fragments and in a central immunodominant epitope [37]. In summary, the interaction of MBP with actin involves the majority of the protein sequences, which effectively masks MBP from the proteasome. Unlike MBP-actin complexes, the interaction of MBP with CaM is less related to its charge: the binding properties of the two MBP charge isoforms—C1 and C8—are very similar [36]. More importantly, MBP contains a distinct CaM-binding segment, which is located near the C-terminus and corresponds to residues 138–156 of human 18.5 kDa MBP. MBP_138–156_ interacts mainly with the C-terminal lobe of CaM, and a conformational change accompanies binding [31]. Thus, the limited surface of protein-protein contact may reduce the ability of CaM to protect MBP from proteasomal hydrolysis.

Relevant data regarding the intracellular proteasome concentration in mammalian cells are lacking; however, the concentration of 26S proteasome in the cytoplasm of yeast is estimated to be 140–200 nM [38]. This concentration is similar to that observed in our* in vitro* assays. CaM is known to interact with a number of target proteins, including myosin light chain kinase, calcineurin, neuronal nitric oxide synthase, and phosphodiesterase. The maximum free Ca^2+^-CaM concentration in HEK 293 cells is only 50–60 nM at resting conditions, while the total available calmodulin concentration (apo-CaM and Ca^2+^-CaM) is 6–10 *μ*M [39, 40]. In turn, the total concentration of actin in nonmuscular cells is typically 2-3 mg/mL (46–70 *μ*M) [41]. Approximately 60% of cellular actin is polymerized, and the rest of the protein is mostly bound to profilin and thymosin-*β*4; the concentration of free monomeric actin is estimated to be 2 *μ*M [42]. Because MBP may bind actin filaments, the intracellular concentration of actin accessible for interaction with MBP may be estimated to be 20–40 *μ*M. Proteasomal MBP degradation was significantly inhibited at actin and CaM concentrations of 10–20 *μ*M. Thus, we suggest that CaM is unlikely to protect MBP from the 26S proteasome* in vivo*, whereas actin is a potential “nanny protein” [43] for MBP.

Proteins and polypeptides that mimic MBP were shown to inhibit proteasomal MBP degradation by competing with it for 26S proteasome binding. Both histone H1.3 and the MS therapeutic agent GA, which mimics MBP in both charge and structure, could inhibit the 26S-mediated MBP proteolysis* in vitro *and* ex vivo*. The deconvolution of the *K*_*D*_ of histone H1.3- and GA-26S proteasome interaction based on the inhibition curves results in values of 1 and 10 *μ*M, respectively (Figures 3(c) and 3(d)). Histone H1.3 protects MBP from proteasomal hydrolysis in a “suicidal” manner, whereas GA seems to be resistant to proteasome-mediated hydrolysis. According to the mechanism of action proposed for GA in MS, this agent acts mainly on the periphery and not in the CNS. Thus, the direct competition of GA with MBP for 26S proteasome binding inside oligodendrocytes seems to be questionable. We further suggest that GA may affect intracellular MBP processing in antigen-presenting cells outside the CNS, especially in the context of the recently reported proteasome-dependent presentation of MHC II-restricted antigens [44].

## 5. Concluding Remarks

In this study, we showed two possibilities to protect MBP from proteasome-mediated hydrolysis in order to compensate lack of control via ubiquitination system. First, 26S proteasome failed to recognize MBP when it is associated with naturally occurring MBP-binding proteins, including but probably not restricted to actin and CaM. Importantly, this interaction, which is characterized by large surface contact and accompanied by neutralization, is evidently more effective than “key-lock” binding. These results suggest that a number of negatively charged proteins that are known to be engaged in protein-protein interactions with MBP may potentially serve as “nanny proteins” that partially defend MBP from intracellular degradation. Second, polypeptides that mimic MBP restrict its access to the 26S proteasome. Further studies should identify possible physiologically relevant basic and intrinsically disordered “gatekeepers” that can protect MBP from proteasome-mediated degradation.

## Figures and Tables

**Table 1:** Panel of tested proteins.

Protein	MW	^ 1^pI
MBP from bovine brain	18.3	11.3
Actin from porcine muscle	41.8	5.2
Ubiquitin human (Ub)	8.6	7.4
BSA	66.4	5.6
Glatiramer acetate (GA)	~7 (5–9)	~10.3
Histone H1.3 human	22.2	11.0
Ub_4_	34.4	6.6
GST human	48.8	~6.5
Apo-calmodulin human (CaM)	16.7	4.1

^1^Isoelectric points of proteins listed in the Table 1 were calculated using ExPASy Compute pI/Mw tool http://web.expasy.org/compute_pi/.

## References

[1] Lisak RP, Zweiman B (1977). In vitro cell-mediated immunity of cerebrospinal-fluid lymphocytes to myelin basic protein in primary demyelinating diseases. *New England Journal of Medicine*.

[2] Carnegie PR, Bencina B, Lamoureux G (1967). Experimental allergic encephalomyelitis. Isolation of basic proteins and polypeptides from central nervous tissue. *Biochemical Journal*.

[3] Ota K, Matsui M, Milford EL, Mackin GA, Weiner HL, Hafler DA (1990). T-cell recognition of an immunodominant myelin basic protein epitope in multiple sclerosis. *Nature*.

[4] Martin R, Howell MD, Jaraquemada D (1991). A myelin basic protein peptide is recognized by cytotoxic T cells in the context of four HLA-DR types associated with multiple sclerosis. *The Journal of Experimental Medicine*.

[5] Jurewicz A, Biddison WE, Antel JP (1998). MHC class I-restricted lysis of human oligodendrocytes by myelin basic protein peptide-specific CD8 T lymphocytes. *Journal of Immunology*.

[6] Huseby ES, Liggitt D, Brabb T, Schnabel B, Öhlén C, Goverman J (2001). A pathogenic role for myelin-specific CD8^+^ T cells in a model for multiple sclerosis. *The Journal of Experimental Medicine*.

[7] Rock KL, Gramm C, Rothstein L (1994). Inhibitors of the proteasome block the degradation of most cell proteins and the generation of peptides presented on MHC class I molecules. *Cell*.

[8] Hershko A, Heller H, Elias S, Ciechanover A (1983). Components of ubiquitin-protein ligase system. Resolution, affinity purification, and role in protein breakdown. *The Journal of Biological Chemistry*.

[9] Tanahasi N, Tsurumi C, Tamura T, Tanaka K (1993). Molecular structures of 20S and 26S proteasomes. *Enzyme and Protein*.

[10] Matyskiela ME, Lander GC, Martin A (2013). Conformational switching of the 26S proteasome enables substrate degradation. *Nature Structural and Molecular Biology*.

[11] Kravtsova-Ivantsiv Y, Cohen S, Ciechanover A (2009). Modification by single ubiquitin moieties rather than polyubiquitination is sufficient for proteasomal processing of the p105 NF-kappaB precursor. *Molecular Cell*.

[12] Shabek N, Herman-Bachinsky Y, Buchsbaum S (2012). The size of the proteasomal substrate determines whether its degradation will be mediated by mono- or polyubiquitylation. *Molecular Cell*.

[13] Murakami Y, Matsufuji S, Kameji T (1992). Ornithine decarboxylase is degraded by the 26S proteasome without ubiquitination. *Nature*.

[14] Sheaff RJ, Singer JD, Swanger J, Smitherman M, Roberts JM, Clurman BE (2000). Proteasomal turnover of p21^Cip1^ does not require p21^Cip1^ ubiquitination. *Molecular Cell*.

[15] Ciechanover A, Stanhill A (2014). The complexity of recognition of ubiquitinated substrates by the 26S proteasome. *Biochimica et Biophysica Acta—Molecular Cell Research*.

[16] Tofaris GK, Layfield R, Spillantini MG (2001). *α*-Synuclein metabolism and aggregation is linked to ubiquitin-independent degradation by the proteasome. *FEBS Letters*.

[17] Tsvetkov P, Reuven N, Prives C, Shaul Y (2009). Susceptibility of p53 unstructured N terminus to 20 S proteasomal degradation programs the stress response. *Journal of Biological Chemistry*.

[18] Belogurov AJr, Kudriaeva A, Kuzina E (2014). Multiple sclerosis autoantigen myelin basic protein escapes control by ubiquitination during proteasomal degradation. *Journal of Biological Chemistry*.

[19] Belogurov AA, Ponomarenko NA, Govorun VM, Gabibov AG, Bacheva AV (2009). Site-specific degradation of myelin basic protein by the proteasome. *Doklady Biochemistry and Biophysics*.

[20] Kuzina ES, Chernolovskaya EL, Kudriaeva AA (2013). Immunoproteasome enhances intracellular proteolysis of myelin basic protein. *Doklady Biochemistry and Biophysics*.

[21] Harauz G, Ishiyama N, Hill CMD, Bates IR, Libich DS, Farès C (2004). Myelin basic protein—diverse conformational states of an intrinsically unstructured protein and its roles in myelin assembly and multiple sclerosis. *Micron*.

[22] Boggs JM, Homchaudhuri L, Ranagaraj G, Liu Y, Smith GS, Harauz G (2014). Interaction of myelin basic protein with cytoskeletal and signaling proteins in cultured primary oligodendrocytes and N19 oligodendroglial cells. *BMC Research Notes*.

[23] Hill CMD, Harauz G (2005). Charge effects modulate actin assembly by classic myelin basic protein isoforms. *Biochemical and Biophysical Research Communications*.

[24] Libich DS, Hill C, Bates IR (2003). Interaction of the 18.5-kD isoform of myelin basic protein with Ca2^+^-calmodulin: effects of deimination assessed by intrinsic Trp fluorescence spectroscopy, dynamic light scattering, and circular dichroism. *Protein Science*.

[25] Majava V, Wang C, Myllykoski M (2010). Structural analysis of the complex between calmodulin and full-length myelin basic protein, an intrinsically disordered molecule. *Amino Acids*.

[26] Smith GST, De Avila M, Paez PM (2012). Proline substitutions and threonine pseudophosphorylation of the SH3 ligand of 18.5-kDa myelin basic protein decrease its affinity for the Fyn-SH3 domain and alter process development and protein localization in oligodendrocytes. *Journal of Neuroscience Research*.

[27] Homchaudhuri L, Polverini E, Gao W, Harauz G, Boggs JM (2009). Influence of membrane surface charge and post-translational modifications to myelin basic protein on its ability to tether the Fyn-SH3 domain to a membrane in vitro. *Biochemistry*.

[28] Miller SD, Karpus WJ, Davidson TS (2010). Experimental autoimmune encephalomyelitis in the mouse. *Current Protocols in Immunology*.

[29] Glickman MH, Rubin DM, Fried VA, Finley D (1998). The regulatory particle of the *Saccharomyces cerevisiae* proteasome. *Molecular and Cellular Biology*.

[30] Bamm VV, Ahmed MAM, Harauz G (2010). Interaction of myelin basic protein with actin in the presence of dodecylphosphocholine micelles. *Biochemistry*.

[31] Majava V, Petoukhov MV, Hayashi N, Pirilä P, Svergun DI, Kursula P (2008). Interaction between the C-terminal region of human myelin basic protein and calmodulin: analysis of complex formation and solution structure. *BMC Structural Biology*.

[32] Teitelbaum D, Aharoni R, Sela M, Arnon R (1991). Cross-reactions and specificities of monoclonal antibodies against myelin basic protein and against the synthetic copolymer 1. *Proceedings of the National Academy of Sciences of the United States of America*.

[33] Munishkina LA, Fink AL, Uversky VN (2004). Conformational prerequisites for formation of amyloid fibrils from histones. *Journal of Molecular Biology*.

[34] Deveraux Q, Ustrell V, Pickart C, Rechsteiner M (1994). A 26 S protease subunit that binds ubiquitin conjugates. *Journal of Biological Chemistry*.

[35] Husnjak K, Elsasser S, Zhang N (2008). Proteasome subunit Rpn13 is a novel ubiquitin receptor. *Nature*.

[36] Wang C, Neugebauer U, Bürck J (2011). Charge isomers of myelin basic protein: structure and interactions with membranes, nucleotide analogues, and calmodulin. *PLoS ONE*.

[37] Ahmed MA, Bamm VV, Shi L (2009). Induced secondary structure and polymorphism in an intrinsically disordered structural linker of the CNS: solid-state NMR and FTIR spectroscopy of myelin basic protein bound to actin. *Biophysical Journal*.

[38] Pack CG, Yukii H, Toh-e A (2014). Quantitative live-cell imaging reveals spatio-temporal dynamics and cytoplasmic assembly of the 26S proteasome. *Nature Communications*.

[39] Black DJ, Tran Q-K, Persechini A (2004). Monitoring the total available calmodulin concentration in intact cells over the physiological range in free Ca^2+^. *Cell Calcium*.

[40] Persechini A, Cronk B (1999). The relationship between the free concentrations of Ca^2+^ and Ca^2+^- calmodulin in intact cells. *The Journal of Biological Chemistry*.

[41] Pollard TD, Blanchoin L, Mullins RD (2000). Molecular mechanisms controlling actin filament dynamics in nonmuscle cells. *Annual Review of Biophysics and Biomolecular Structure*.

[42] Kiuchi T, Nagai T, Ohashi K, Mizuno K (2011). Measurements of spatiotemporal changes in G-actin concentration reveal its effect on stimulus-induced actin assembly and lamellipodium extension. *Journal of Cell Biology*.

[43] Tsvetkov P, Reuven N, Shaul Y (2009). The nanny model for IDPs. *Nature Chemical Biology*.

[44] Tewari MK, Sinnathamby G, Rajagopal D, Eisenlohr LC (2005). A cytosolic pathway for MHC class II-restricted antigen processing that is proteasome and TAP dependent. *Nature Immunology*.

